# Identification of Blueberry miRNAs and Their Targets Based on High-Throughput Sequencing and Degradome Analyses

**DOI:** 10.3390/ijms19040983

**Published:** 2018-03-26

**Authors:** Guangping Li, Yun Wang, Xiaoming Lou, Hailing Li, Changqing Zhang

**Affiliations:** 1 College of Forest, Nanjing Forestry University, Nanjing 210037, China; liguangping108@sina.com; 2 College of Horticulture, Jinling Institute of Technology, Nanjing 210038, China;wangyun@jit.edu.cn(Y.W.); lihailing@jit.edu.cn (H.L.); 3 College of Horticultural Technology, Suzhou Polytechnic Institute of Agriculture, Suzhou 215008, China; louxiaoming@aliyun.com

**Keywords:** blueberry, miRNAs, RNA-Seq, gene expression, degradome analysis

## Abstract

miRNAs are important regulators of plant gene expression. To better characterize their functions, we applied high-throughput sequencing and degradome analyses to investigate three blueberry (*Vaccinium ashei*) tissues. A total of 127 known and 101 novel miRNAs were identified. Moreover, 141 targets for 42 known and 19 novel miRNAs were experimentally validated by degradome sequencing. A functional analysis of these miRNA targets revealed they were associated with diverse biological activities and several pathways, e.g., anthocyanin biosynthesis and cytokinin signal transduction. The data presented herein expand our understanding of the regulation of blueberry miRNAs during floral and fruit development stages. They may also provide new insights into the roles of miRNAs during anthocyanin biosynthesis in blueberry fruits.

## 1. Introduction

miRNAs are important small RNAs (sRNAs) that are generated from single-stranded RNA precursors capable of forming hairpin structures. miRNAs can regulate cellular and metabolic processes by binding and cleaving mRNA or suppressing translation [1]. Blueberry is an economically important fruit crop. Its fruit is rich in anthocyanins, which are reportedly beneficial to human health because of their ability to improve night vision, retard tumor growth, and reduce the risk of cardiovascular and neurodegenerative diseases, among other effects [2,3,4,5]. However, our understanding of the regulation of blueberry miRNAs remains very limited.

Recently, using next-generation sequencing technology, a total of 412 conserved miRNAs and 35 predicted novel miRNAs have been identified from blueberry fruits [6]. The potential target genes of many of these miRNAs were also predicted to be transcription factors and enzymes. They were involved in many important biological processes, such as anthocyanin biosynthesis. Yet, whether these targets are overstated, and the interactions between a given miRNA and its targets are actual are still uncertain. Therefore, it is necessary to further perform a genome-wide experimental validation, which will contribute to a better understanding of the regulation of blueberry miRNAs. More recently, another study contributing to this motivation has been performed [7]. It analyzed fruit ripening-related miRNAs and their targets, and validated 178 targets for 41 known and 7 novel miRNAs based on degradome analyses. However, it is still insufficient for us to perfectly understand the regulation of blueberry miRNAs based on the amount of known miNRAs identified in a relatively sophisticated plant, Arabidopsis [8].

Transcriptome sequencing has been used to assemble transcript sequences and analyze gene expression [9,10,11], and sRNA sequencing has been used for the genome-wide identification of miRNAs, including new, low-abundance miRNAs as well as the conserved, highly abundant miRNAs [6]. Meanwhile, degradome sequencing has been applied to identify miRNA target genes at the genome-wide level [12,13]. Combining the data derived from transcriptome, sRNA, and degradome sequencing experiments represents an alternative strategy for the systematic analysis of miRNA regulation on a genome-wide level [14].

In this study, we identified blueberry miRNAs and their targets in flowers, young fruits, and ripe fruits via the bioinformatics-based analysis of transcriptome, sRNA, and degradome sequencing data. We identified 127 known and 101 potentially novel miRNAs. Moreover, 141 miRNA targets were confirmed by degradome sequencing. Of these targets, three encoded the enzymes or transcription factors related to anthocyanin biosynthesis, and one affected cytokinin signal transduction.

## 2. Results

### 2.1. Small RNA Profiles in Blueberry

Sequencing the sRNA libraries for *V. ashei* flowers, young fruits, and ripe fruits resulted in 12.1, 12.2, and 11.2 million raw reads, respectively (Table S1). The removal of adaptor sequences, junk reads, reads shorter than 18 bp, rRNA, snRNA, snoRNA, and tRNA produced 11.9, 12.1, and 10.8 million clean reads, corresponding to 6.6, 7.0, 6.0 million unique reads for flowers, young fruits, and ripe fruits, respectively. The clean reads accounted for about 96% of the raw reads, which suggested a useful group of sRNAs was obtained with a reasonable sequencing depth.

### 2.2. Identification of Known miRNAs

To identify *V. ashei* miRNAs, all clean sRNAs were compared with known mature plant miRNAs in the miRBase 21 database. We identified 24 miRNAs with no mismatches as well as 103 miRNAs with at least a 16-nt overlap with known miRNAs to consider the likelihood that there are differences in the miRNA sequences of diverse species [15] (Table S2).

Among the 24 miRNAs that perfectly matched known miRNAs, vas-miR166a, vas-miR166g-3p, vas-miR535d, and miR168a-5p were highly expressed in all three analyzed tissues, with more than 1000 reads per million (RPM). A different expression analysis indicated that the expression levels of some miRNAs were higher or lower expressed in specific tissues. For example, miR390a-5p expression was higher in flowers, while miR157a-5p expression levels were lower in young fruits and miR167a expression levels were lower in ripe fruits. Moreover, we also observed that miR-5p miRNAs were more abundant than miR-3p miRNAs in vas-miR168a.

Of the aforementioned 103 miRNAs that overlapped with known miRNAs, two miRNAs (vas-miR6149a and vas-miR156) were detected with more than 1000 RPM, while 12 miRNAs were observed with less than 10 RPM in all three tissues. Among the variants with more than 100 RPM, the expression of vas-miR8175 was higher in flowers; vas-miR156, vas-miR858b, vas-miR5077, and vas-miR7532a expression levels were higher in ripe fruits; and vas-miR7122a, vas-miR8175, and vas-miR408b-5p expression levels were lower in young fruits.

### 2.3. Identification of Novel miRNAs

To identify novel miRNAs, all non-annotated sRNAs were mapped to *V. ashei* unigenes. Hairpin structures were predicted using the Mireap program. We identified 101 candidate miRNAs that were 20–23 nt long, including six that were 20 nt long, 63 that were 21 nt long, 12 that were 22 nt long, and 20 that were 23 nt long (Table S3). The precursors comprised 54–363 nt, with an average of 150 nt (Table S3). Additionally, the minimum free energy for their hairpin structures was less than −18.2 kcal/mol, suggesting the secondary structures were stable.

Most of the novel miRNAs were detected at low levels, with none at more than 1000 RPM. Only four miRNAs (vas-miR-14, vas-miR-20, vas-miR-25, and vas-miR-46) were observed with more than 100 RPM in all three libraries. Among the novel miRNAs that accumulated to more than 50 RPM, vas-miR-58 and vas-miR-93 were specific to young and ripe fruits, respectively. Additionally, vas-miR-35 expression was expressed higher in ripe fruits, while vas-miR-32 and vas-miR-45 exhibited lower expression in flowers and young fruits, respectively.

The vas-miR-41 precursors could be bidirectionally transcribed because the genes comprised a palindrome structure. The sense and antisense transcripts formed the same hairpin structure. Similar findings were reported for soybean [16], switchgrass [17], and panax ginseng [18].

We identified 15 miRNA–miRNA* pairs. Moreover, there were more miRNAs from the 5′ arm of the hairpin structure than from the 3′ arm, which was similar to the results for known miRNAs. Moreover, all miRNAs* were expressed at relatively low levels, with none accumulating more than 10 RPM. Furthermore, their tissue specificities were consistent with those of their miRNAs.

### 2.4. Identification of miRNA Targets Based on a Degradome Analysis

To characterize the regulatory roles of blueberry miRNAs, the target genes of all detected miRNAs were identified by analyzing the degradome sequencing data.

A total of 20.6, 18.7, and 17.6 million tags were obtained for flowers, young fruits, and ripe fruits, respectively. After removing the adaptor tags, low-quality tags, rRNA, snRNA, snoRNA, and tRNA, approximately 20.3, 18.4, and 17.4 million clean tags remained for flowers, young fruits, and ripe fruits, respectively. Mapping degradome tags to the *V. ashei* unigenes and a subsequent analysis using the CleaveLand pipeline resulted in the identification of 141 target genes for 42 known and 19 novel miRNAs (Table S4). Of these targets, 53, 53, and 65 were for flowers, young fruits, and ripe fruits. Additionally, 11 targets were common to 2 tissues, while 9 were common to all 3 tissues.

**Figure 1:**
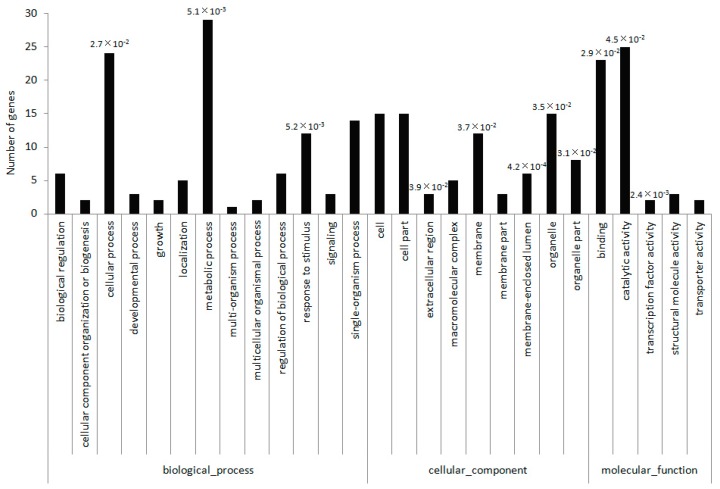
Gene ontology classification of blueberry miRNA target genes. Number above the bar indicatesthe *p*-value of target genes enriched in functional category. Only the enrichments with*p*-values less than 0.05 were showed.

To further gain insights into the common biological functions, we conducted a GO analysis on all miRNA target genes, which resulted in the annotation of 50 miRNA target genes with GO terms. The annotations covered a wide range of biological activities (Figure 1). These included 41 genes involved in 13 biological processes, 18 genes related to 9 cellular components, and 39 genes involved in 5 molecular functions. Metabolic process (GO: 0008152), catalytic activity (GO: 0003824), and cellular process (GO: 0009987) were the top three terms, with 29, 24, and 23 target genes, respectively. Their *p* values of enrichment, calculated on basis of hypergeometric distribution by using whole genome as background, were 5.1 × 10^−3^, 2.7 × 10^−2^, and 4.5 × 10^−2^, respectively, indicating that cellular metabolic processes, biologically catalyzed reactions, and cell physiological processes are the typical activities regulated by miRNAs in *V. ashei* flowers and fruits. Additionally, some of target genes were also significantly enriched in function categories, response to stimulus(GO:0050896) and transcription factor activity(GO:0000988). Although their numbers were the highest, those results provide clues to further study functions of miRNA-target regulatory pathway.

**Figure 2:**
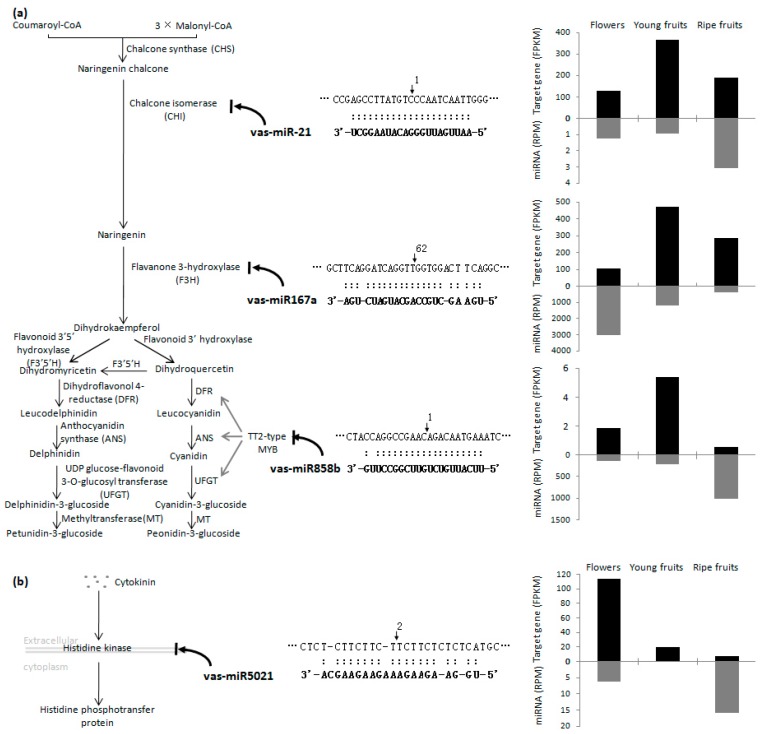
Blueberry miRNA–target gene regulatory networks involved in anthocyanin biosynthesis (**a**) and cytokinin signal transduction (**b**). The right of each miRNA showed the corresponding miRNA:target alignment and the expression levels of miRNA and its target gene in flowers, young fruits, and ripe fruits. The arrow on the target transcript indicates the cleavage site and the number above the alignment indicates the number of RNA degradation tags with the 5′-end overlapped to the cleavage site.

To further elucidate the miRNA–target gene regulatory networks, we mapped miRNA target genes to KEGG (kyoto encyclopedia of genes and genomes) pathways. Interestingly, several important pathways related to floral and fruit development or fruit quality were included. The first is the anthocyanin biosynthesis pathway (Figure 2a). Three miRNAs and their target genes were identified in the present study. The first one was vas-miR-21. Its expression levels were 0.90 and 3.08 RPM in young and ripe fruits, respectively. Its target was a gene encoding chalconeisomerase (CHI), which was expressed at 365.60 and 191.28 FPKM (fragments per kilo base of transcript per million fragments mapped) in young and ripe fruits, respectively. Our degradome analysis revealed that the CHI transcript was cleaved by vas-miR-21 in ripe fruits, but not in young fruits. The second identified miRNA was vas-miR167a. Our degradome analysis confirmed that vas-miR167a cleaved the transcript of flavanone 3-hydroxylase(F3H) in young fruits. Its expression levels were 1180.38 and 369.56 RPM in young and ripe fruits respectively, while F3H transcripts accumulated to 469.73 and 286.66 FPKM in young and ripe fruits respectively. It indicated F3H expression might also be regulated by another unknown mechanism. The third miRNA was vas-miR858b, which was expressed at 217.02 and 1017.67 RPM in young and ripe fruits, respectively. Its target was a transcription factor gene encoding a TT2 (transparent testa 2)-type MYB (v-myb avian myeloblastosis viral oncogene homolog). Our degradome analysis indicated the transcript was cleaved by vas-miR858b in young and ripe fruits. Moreover, the transcript encoding the TT2-type MYB accumulated to 5.35 and 0.55 FPKM in young and ripe fruits, respectively. The second is cytokinin signal transduction pathway. As indicated in Figure 2b, a gene encoding a histidine kinase was identified as a target of vas-miR5021 in ripe fruits. The vas-miR5021 expression levels were 0 and 16.37 RPM in young fruits and ripe fruits, respectively. Meanwhile, the target gene transcript levels were 20.91 and 8.40 FPKM in young fruits and ripe fruits, respectively. Additionally, a gene encoding protein phosphatase 2C (PP2C), which is a central component of abscisic acid (ABA) signaling pathway [19], was identified as a target of vas-miR-74 (Table S4),and a gene encoding pectinesterase involved in pentose and glucuronate interconversions was identified as the target of vas-miR-74, vas-miR-98, and vas-miR168a-5p (Table S4). Figure S1 displaysall the identified miRNA–target gene regulatory networks generated from the software of cytoscape [20].

## 3. Discussion

The miRNAs are important for plant development and responses to biotic and abiotic stresses. The development of high-throughput sequencing technology has led to the identification of miRNAs and their targets in many plant species. However, there is very limited information available regarding miRNAs and their targets in blueberry, which is an economically important fruit crop. Thus, we completed high-throughput sequencing and degradome analyses to comprehensively characterize the regulation of miRNAs during the development of blueberry flowers and fruits.

**Figure 3:**
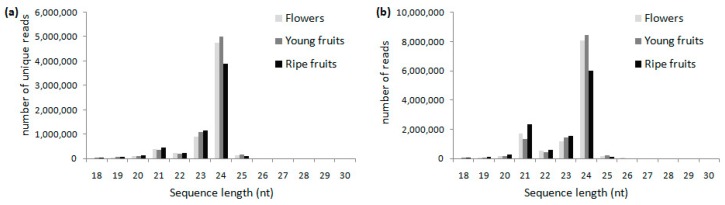
Length distribution of unique sRNAs (**a**) and clean reads (**b**) for *V. ashei* flowers, young fruits, and ripe fruits.

Sequencing the sRNA libraries for *V. ashei* flowers, young fruits, and ripe fruits resulted in 11.9, 12.1, and 10.8 million clean reads, respectively. Read lengths for the three samples exhibited a similar distribution pattern, and ranged from 18 to 30 nt. The majority of unique sRNAs were 21–24 nt long (Figure 3a), corresponding to the typical size for dicer-derived products. The most abundant sRNAs were 24 nt long. The result was consistent with those of previous studies involving potato [21], citrus trifoliata [22],durum wheat [23], and rice [24]. However, it was in contrast to the data obtained for grapevine [12] and tomato [25], in which the predominant sRNAs were 21 nt long. An analysis of the distribution of clean read lengths also revealed the most common length was 24 nt (Figure 3b). These observations suggested 24-nt sRNAs may be particularly important in blueberry flowers and fruits. Our results were also indicative of the complexity of the blueberry genome as the 24nt sRNAs were mainly siRNAs associated with repressive chromatin [1].

Cross-species comparisons of miRNAs have identified 29 conserved miRNA families in fruit trees [26]. These families can be detected in at least three of the following four fruit tree species: peach, apple, citrus, and grapevine. We detected 21 of the 29 families in the current study. This indicates these miRNAs are also conserved in blueberry. Further compared with the two datasets of blueberry miRNAs published respectively by Yue [6] and Hou [7], a total of 61 miRNAs were shared with one of Yue’s and Hou’s in our miRNAs (Table S5). Excepting these, there are still about 73% new miRNAs reported. This implicated, the number of blueberry miRNAs might be not close to saturation to date, on the one hand. On the other hand, it also implicated that there might be false positive miRNAs in published data. The study of blueberry miRNAs still has a long way to go.

Our degradome analysis has resulted in the identification of 141 target genes for 44 known and 19 novel miRNAs. GO analysis revealed that genes related to the metabolic process, catalytic activity, cellular process categories, response to stimulus, transcription factor activity, etc. were the main miRNA targets in blueberry flowers and fruits. Earlier studies examining the blueberry transcriptome or expressed sequence tags also concluded that genes associated with metabolic processes, catalytic activities, and cellular processes were actively expressed [6,10,27]. These results provide clues to further study functions of miRNA-target regulatory pathway.

Blueberry fruits are a rich source of anthocyanins. The TT2-type MYBs can promote anthocyanin accumulation in diverse fruits by binding to the promoters of structural genes to regulate expression [28,29]. In apple, a gene encoding MdMYB9 is a predicted target of mdm-miR858, and the MdMYB9 and miR858 expression levels reportedly exhibit the opposite trends [30]. Similarly, we identified a gene encoding a TT2-type MYB as the target of vas-miR858b. Moreover, vas-miR858b and the TT2-type MYB gene were differentially expressed. Specifically, vas-miR858b expression levels were 217.02 and 1017.67 RPM in young and ripe fruits, respectively, while the TT2-type MYB transcript levels were 5.35 and 0.55 FPKM in young and ripe fruits, respectively. This further confirms that vas-miR858b negatively regulates the accumulation of anthocyanin in blueberry fruits, which implicated that anthocyanin biosynthesis might be very week in fully mature fruit. Interestingly, we identified the CHI and F3H structural genes as the targets of vas-miR-21 and vas-miR167a, respectively. A previous study revealed that miR167a targets an IAA amidohydrolase gene, IAR3, as part of a drought tolerance mechanism [31]. The higher accumulation of transcripts in young fruits suggested CHI was more actively expressed in developing fruits than in ripe fruits. Our degradome analysis revealed that the CHI transcript was cleaved by vas-miR-21 in ripe fruits, but not in young fruits. This further indicated CHI expression was repressed in ripe fruits, which was consistent with the lower accumulation of CHI in ripe fruits than in young fruits. While positive correlation between expression levels of F3H and vas-miR167a implicated that F3H expression might also be regulated by another unknown mechanism.

Cytokinin is an essential hormone for nearly all aspects of plant growth and development. Cytokinin activities progressively decline as fruits mature [32], implying there exists a mechanism that gradually inactivates cytokinin signal transduction. We identified a gene encoding a histidine kinase as a target of vas-miR5021 in ripe fruits. Histidine kinase is a cytokinin receptor that can transduce cytokinin signals across the plasma membrane. Our sequencing results confirmed that the transcript abundance for the histidine kinase gene decreased as young fruits ripened, while vas-miR5021 expression significantly increased during the same period. This further supports the likelihood of a mechanism that inactivates cytokinin signal transduction in ripening fruits.

Abscisic acid is a central regulator of fruit ripening. Its content increases markedly to initiate blueberry fruit ripening, and peak levels occur at the ~75% blue stage [27]. In our study, PP2C expression exhibited a similar pattern, with the corresponding transcript levels reaching 40.61 and 290.06 FPKM in young and ripe fruits, respectively. An earlier study involving beechnut indicated that PP2C expression levels in seeds and young seedlings were higher expressed by exogenously applied ABA. However, the over-expression of PP2C in transgenic *A. thaliana* decreased ABA sensitivity in germinating seeds and in response to different abiotic stresses [33]. Additionally, PP2C has been confirmed as a negative regulator of ABA signaling. In the present study, PP2C was identified as a target of vas-miR-74 in ripe blueberry fruits. The vas-miR-74 expression levels were 0 and 0.81 RPM in young and ripe fruits, respectively, which is consistent with the PP2C expression levels. In a previous study, PP2C was identified as a target of cand_mir_322-3p in the ABA signaling network [34], a target of ttu-miR008 and tae-miR408 in wheat [23,35], and a target of five miRNAs—including sly-miR159b-3p, sly-miR393a, sly-miR268, sly-miR6020a-5p, and sly-miR7696a-3p—in tomato [36].

## 4. Materials and Methods

### 4.1. Plant Materials

Eleven-year-old rabbiteye blueberry plants (*V. ashei* cultivar ‘Powderblue’) from cutting propagation were grown at the experimental farm of the Jinling Institute of Technology. Blooming flowers, young fruits (i.e., expanding, approximately 5 mm diameter), and ripe fruits were collected from five trees with similar growth. For each sample type, we collected 100g tissues from each tree, and pooled them uniformly, and further transported them to the laboratory on dry ice. They were then frozen in liquid nitrogen prior to the extraction of total RNA using a standard CTAB method.

### 4.2. Construction and Sequencing of Transcriptome, sRNA, and Degradome Libraries

Transcriptome cDNA, sRNA, and degradome cDNA libraries were constructed and sequenced for flowers, young fruits, and ripe fruits. In which three biological replicates were used, and a total of three transcriptome libraries, three small RNA libraries, and three degradome libraries were produced and used in our analysis. When constructing the transcriptome cDNA library, poly-(A) mRNAs were isolated using oligo-(dT) beads, after which the cDNA library was prepared using an Illumina kit according to manufacturer’s recommendations. The library was sequenced using the HiSeq™ 4000 platform (The Beijing Genomics Institute, ShenZhen, China). Regarding the sRNA library, 18–30 ntsRNA fragments were isolated using 15% denaturing polyacrylamide gel electrophoresis and then ligated to 3′ and 5′ adaptors for a subsequent conversion to cDNA by a reverse transcription polymerase chain reaction. The library was sequenced using the Genome Analyzer (The Beijing Genomics Institute, ShenZhen, China). To construct the degradome library, mRNA was used to anneal with biotinylated random primers, and then, strapavidin capture of RNA fragments was performed using biotinylated random primers. Finally, a 5′ adaptor ligation was only performed to those RNAs containing 5′-monophosphates, followed with reverse transcription and PCR. The resulting library underwent single-end sequencing using the HiSeq™ 4000 platform. The sequence data have been submitted to China National GenBank(https://db.cngb.org/cnsa/) under accession no. CNP0000007 for the transcriptome sequences, CNP0000008 for the sRNA sequences, and CNP0000009 for the degradome sequences.

### 4.3. Analysis of Transcriptome Sequencing Data

Adaptor sequences, low-quality reads, and empty reads were removed from the raw reads to obtain clean transcriptome sequencing reads, which were then assembled into unigenes using the Trinity short-reads assembling program. All unigenes for flowers, young fruits, and ripe fruits were joined and redundancies were eliminated with sequence clustering software to acquire non-redundant unigenes. Finally, we conducted a blastx search of protein databases (e.g., NT, NR, Swiss-Prot, KEGG, and COG) using the unigenes as queries. The best alignments were used to determine the sequence direction and annotate unigenes. The Blast2GO program (www.blast2go.com) was subsequently used to functionally annotate the unigenes with Gene Ontology (GO) terms.

### 4.4. Analysis of sRNA Sequencing Data

Clean sRNA sequencing reads were obtained by removing low-quality sequences, adaptor sequences, junk reads, and reads shorter than 18 bp from raw reads. The Bowtie program was used to align clean reads to the *V. ashei* unigenes obtained from the transcriptome sequencing data. The rRNA, tRNA, snRNA, snoRNA, and other repeated sequences were removed based on searches of the NCBI GenBank and Rfam 11.0 databases. Known miRNAs were detected with Bowtie [23]. First, considering the difference among species, we align clean data to the mature miRNA of all plants in miRBase 21 allowing at least 16 nt overlap; second, we choose the highest expressed sequence for each miRNA family and regard it as a temporary miRNA template; third, we align clean data to the temporary template allowing two mismatches and free gaps. The expression of miRNA is generated by summing the count of aligned reads, and a cutoff of abundance over 2 RPM in at least two libraries is used; fourth, we predict the precursor of the identified miRNA, of which, if there is not a hairpin structure, it will be regarded as pseudo-miRNA. Furthermore, a different expressed analysis of miRNA were also performed by using the method of Expdiff [37]. Considering the lack of biological replicates and technical duplicates in sequencing, a fold-change (log2) threshold of greater than 1 or less than −1 was considered as an indication of significant change [38]. New candidate miRNAs were identified first by mapping unique sRNAs to the *V. ashei* unigenes and then by folding the flanking sequences using the Mireap program (The Beijing Genomics Institute, ShenZhen, China).

### 4.5. Analysis of Degradome Sequencing Data

Clean degradome sequencing tags were obtained by removing low-quality and adaptor sequences from raw tags. The clean tags were aligned to *V. ashei* unigenes using the Bowtie program with no mismatches allowed. The rRNA, tRNA, snRNA, snoRNA, polyN, and other repeated sequences were removed based on searches of the NCBI GenBank and Rfam 11.0 databases. The miRNA target genes were identified by mapping the degradome tags to the *V. ashei* unigenes and conducting an analysis using the CleaveLand pipeline with standard criteria for target cleavage, including a *p* value cut-off of 0.05. The target transcripts were classified into five categories based on the following criteria: Category 0—more than one tag at the cleavage site, with the abundance at the cleavage site equal to the maximum on the transcript, and only one maximum on the transcript; Category 1—more than one tag at the cleavage site, with the abundance at the cleavage site equal to the maximum on the transcript, and more than one maximum on the transcript; Category 2—more than one tag at the cleavage site, with the abundance at the cleavage site less than the maximum, but higher than the median for the transcript; Category 3—more than one tag at the cleavage site, with the abundance at the cleavage site equal to or less than the median for the transcript; Category 4—only one tag at the cleavage site.

## 5. Conclusions

In conclusion, we identified 127 known and 101 potentially novel miRNAs as well as 141 their target genes in blueberry based on high-throughput sequencing and degradome analyses. Our findings also suggest that the miRNA–target gene regulatory networks are related to sulfur metabolism, hormone signal transduction, and anthocyanin biosynthesis, among other processes. The data presented herein may clarify the regulation of blueberry miRNAs, and provide new insights into the effects of miRNAs on the development of blueberry flowers and fruits.

## Supplementary Materials

Supplementary materials can be found at http://www.mdpi.com/1422-0067/19/4/983/s1.
