# Supplementary figures and images for: Identification of Blueberry miRNAs and Their Targets Based on High-Throughput Sequencing and Degradome Analyses
Source: Int J Mol Sci. 2018 Mar 26;19(4):983. doi: 10.3390/ijms19040983 (PMC5979386; doi:10.3390/ijms19040983)

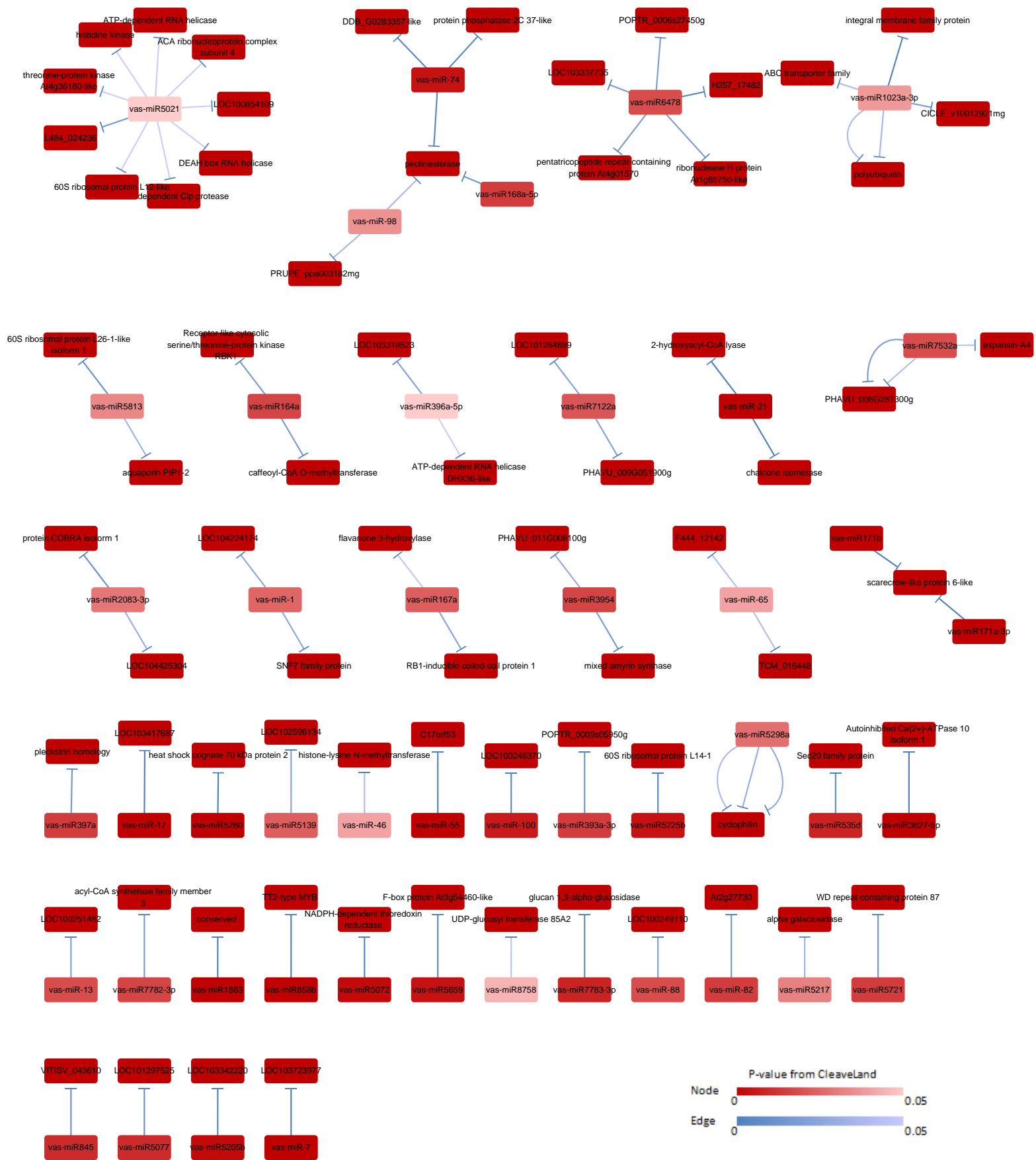

Figure S1. Visualization of the identified miRNA – target gene regulatory networks

Supplement: Supplementary file 1 [file ijms-19-00983-s001.zip › ijms-260334-supplementary/Figure S1.pdf]
